# Trends, Patterns, and Treatment Outcomes of Cancer Among Older Patients in Jordan: A Retrospective Analysis of National Cancer Registry and Institutional Outcome Data

**DOI:** 10.1200/GO.20.00044

**Published:** 2020-05-21

**Authors:** Hikmat Abdel-Razeq, Asem Mansour, Rayan Bater

**Affiliations:** ^1^ Department of Medicine, King Hussein Cancer Center, Amman, Jordan; ^2^ School of Medicine, University of Jordan, Amman, Jordan; ^3^ Department of Radiology, King Hussein Cancer Center, Amman, Jordan

## Abstract

**PURPOSE:**

Cancer is a leading cause of morbidity and the second leading cause of mortality in Jordan and worldwide. Because of their age and comorbidities, older patients may receive suboptimal cancer therapy. This article addresses trends in cancer incidence and reports key treatment outcomes in this age group.

**MATERIALS AND METHODS:**

This is a retrospective study using data obtained from the national Jordan Cancer Registry (JCR) and our institutional cancer registry. The first data set reports only on demographics, whereas the second data set reports also on treatment outcomes. Older patients were defined as those age 65 years or older at time of diagnosis.

**RESULTS:**

Between 2001 and 2015, a total of 19,397 older patients were diagnosed with cancer, representing 29.8% of the total 65,050 patients with cancer diagnosed during this time. More men than women developed cancer, and colorectal, breast, lung, prostate, and bladder cancers were the most commonly reported cancers. Among this age group over the 15-year study period, cancer diagnoses increased by a rate of 77%, much higher than the 55% increment among all ages during the same study period. The 5-year survival rate for all of the 3,821 older patients diagnosed, treated, and followed up at our institution was 33% but varied by stage (63% for stage I disease and 14% for stage IV disease).

**CONCLUSION:**

Cancer diagnoses among older patients are increasing at a rate higher than that of all ages and much higher than the witnessed increase in Jordanian population in same age group, which highlights the importance of looking for factors other than just aging to explain this increase. Strategies to offer better care for this rapidly expanding group are highly needed.

## INTRODUCTION

Cancer is one of the leading causes of morbidity and mortality in Jordan. Latest national mortality data showed that cancer accounts for 16.2% of all deaths, second only to cardiovascular disease, which accounts for 36.7% of the total national mortality.^1^

**CONTEXT**

**Key Objective**
This article addresses trends in cancer incidence and reports key treatment outcomes in older patients in Jordan, a low-income country.
**Knowledge Generated**
Over the 15-year study period, the number of reported cancers in patients age 65 years or older increased by 77% and now accounts for almost 30% of the country’s cancer load. Five-year overall survival for older patients was 33% but varied with the disease stage at presentation, ranging from 14% to 63%.
**Relevance**
Findings reflect the current status of cancer among older patients in low-income countries, such as Jordan. This study also highlights the need for proper planning and strategies to better care for the expected increase in cancer diagnoses among this older age group. Investing in geriatric medicine and palliative and hospice care is highly needed.

Jordan, a Middle Eastern country, occupies an area of 92,000 km^2^.^2^ More than 80% of the population is urban, with almost two thirds of the population residing in the central region and only 10% of the population living in the southern part of the country.^3^ According to the World Bank’s classification, Jordan was recently elevated to classification as an upper-middle-income country.^4^ However, Jordan continues to suffer from high inflation and unemployment rates, insufficient supplies of water, and lack of natural resources.^5^ In 2018, the gross domestic product was estimated at $42.3 billion with an annual growth of < 2.0% per year.^6^

Age is the greatest risk factor for developing cancer; such risk increases significantly after age 50 years. According to the most recent statistical data from National Cancer Institute SEERS Program, the median age at cancer diagnosis is 66 years but varies according to cancer type (eg, 61 years for breast cancer, 66 years for prostate cancer, 68 years for colorectal cancer, and 70 years for lung cancer).^7^

As a result of the aging of the world population and the increasing burden of cancer across all nations and ethnic groups, the field of geriatric oncology is evolving rapidly and has become a focused subspecialty. However, this is occurring at a much slower pace in developing countries.^8^

Care of older patients with cancer is a challenge; declines in physiologic reserve occur with aging, even among those without medical problems. Such factors should be considered when offering various anticancer therapies.

Obviously, planning requires clear understanding of population demography and available resources. In 2015, the total population of Jordan was 9.4 million; 70% of the population (6.5 million) were Jordanians.^9^ Assuming the same growth rate and other socioeconomic variables, these numbers are expected to increase to a population total of 12.9 million (9.0 million Jordanians) in 2030 and 19.0 million (13.1 million Jordanians) in 2050. Currently, 344,000 of all residents (80% Jordanians) are age 65 years or older, representing < 4.0% of the total population. However, these numbers are expected to increase to 491,000 in 2030 and 1.1 million in 2050, representing 5.8% and 10.1%, respectively, of the projected populations.^10^

Life expectancy at birth for the world has increased from 64.2 years in 1990 to 72.6 years in 2019 and is expected to increase further to 77.1 years in 2050. However, large gaps continue to exist between countries. In 2019, life expectancy at birth in some countries lagged 7.4 years behind the global average. High child and maternal mortality, violence, and the continuing impact of the HIV epidemic are among the reasons.^11^ However, none of these problems are important factors in our country. In Jordan, data show that life expectancy at birth is 72.87 years for males and 74.27 years for females. These figures are expected to increase to 74.87 years in males and 76.27 years in females in 2030 and to 75.37 and 76.77 years, respectively, in 2050.^11^

In this article, we use data from the Jordan Cancer Registry (JCR) to study trends in cancer incidence and use our institutional cancer registry to report key treatment outcomes in this age group. Findings should help us understand and better prepare for the increasing number of older patients with cancer that our health care system will face in the near future.

## MATERIALS AND METHODS

This is a retrospective study using data collected and made available by the JCR, which is a population-based registry that was established in 1996 under the jurisdiction of the Ministry of Health and gets published annually; the last published report was in 2015.^12^ Older patients with cancer are defined as those age 65 years or older at time of diagnosis.

We identified all malignancies reported by the JCR among older patients for the period from 2001 to 2015. Because the JCR does not report treatment outcomes or survival data, we also used the data collected by our institutional cancer registry, which is a comprehensive database that was started in 2006 and collects detailed data including treatment modalities and outcomes. This registry represents almost two thirds of the total national patients with cancer and is matched to the data set used to generate the JCR reports. The patients registered in our cancer database have a central pathology review and long-term follow-up in the center including an expanded survivorship program. Permission to publish institutional registry outcome data was obtained from the hospital administration and the scientific and research office.

### Statistical Analysis

Descriptive statistics are provided for all variables. Results for continuous variables are expressed as medians with interquartile ranges. Categorical variables are expressed as numbers and percentages. The Kaplan-Meier method was used to estimate overall survival curves, and patients’ survival times between stage groups were compared using the log-rank test. Overall survival was calculated as the time from primary diagnosis to death from any cause or to the last contact based on the patient’s status (dead or alive). A significance level of *P* ≤ .05 was used in the analysis. All analyses were performed using SAS software version 9.4 (SAS Institute, Cary, NC).

## RESULTS

**FIG 1:**
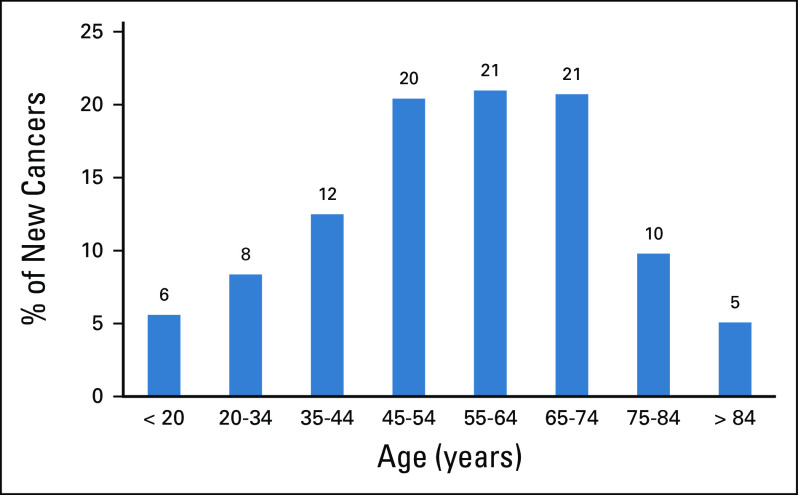
Distribution of new cancers by age group.

According to the latest published annual report by the JCR, the median age at cancer diagnosis was 56 years, with considerable variation according to the cancer site and sex (59 years for men and 52 years for women).^13^ Figure 1 illustrates distribution of new cancers by age group as reported by the JCR in its latest report.^13^ In 2015, the crude incidence rate of all cancers among Jordanians was 84.5 per 100,000 (82.4 for males and 86.4 for females). The age-standardized rate adjusted to the world standard population was 132.4 per 100,000 population (127.8 per 100,000 for males and 136.9 per 100,000 for females).^13^

Over the 15-year study period, a total of 19,397 older patients were diagnosed with cancer in Jordan. This represents 29.8% of the total 65,050 patients diagnosed with cancer during this period. Males were more often diagnosed with cancer than females, with male-to-female ratio of 1.4:1.0. This male predominance was evident across all age subgroups and is mostly a result of the high percentage of prostate, bladder, and lung cancers among men.

**FIG 2:**
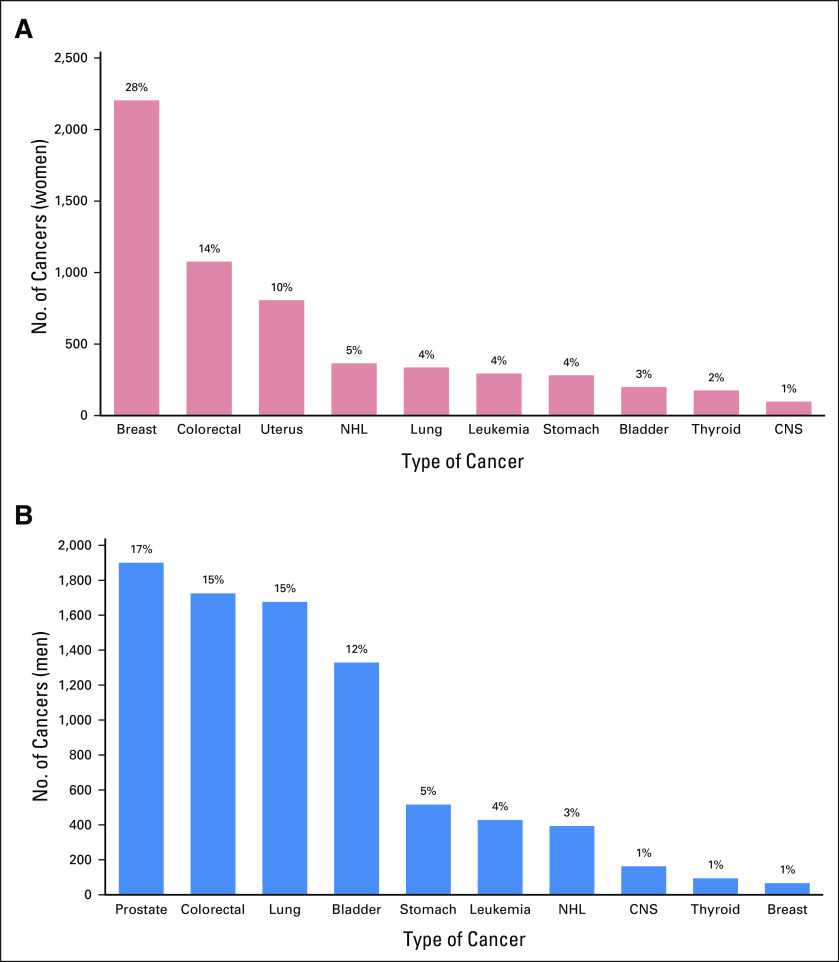
Common cancer sites in (A) female and (B) male older patients. Percentages from total number of women (n = 7,970) and men (n = 11,427). NHL, non-Hodgkin lymphoma.

Lung, prostate, and colorectal cancers were the most common cancers among men, whereas breast, colorectal, and uterine cancers were the most common cancers among women (Figs 2A and 2B). Over the span of 15 years, colorectal cancer was the most frequently reported cancer in both sexes, with a total of 2,802 patients representing 14.4% of cancer burden in the older age group and 37.7% of all colorectal cancers among all age groups. Breast cancer in Jordan, and the Middle East region in general, affects younger patients. The median age at diagnosis is 52 years, almost 10 years younger than in Western societies.^13,14^ During the study period, a total of 2,270 breast cancers were reported among the older patients, representing only 17.9% of the total 12,696 breast cancers reported in all age groups.

Almost three quarters of all prostate cancers (73.4%) were diagnosed among older patients. Bladder, lung, colorectal, and stomach cancers are among the commonly encountered cancers in older patients, representing 49%, 44%, 38%, and 37%, respectively, of cancers in older patients. Table 1 lists the common cancers among both older men and women.

**TABLE 1:**
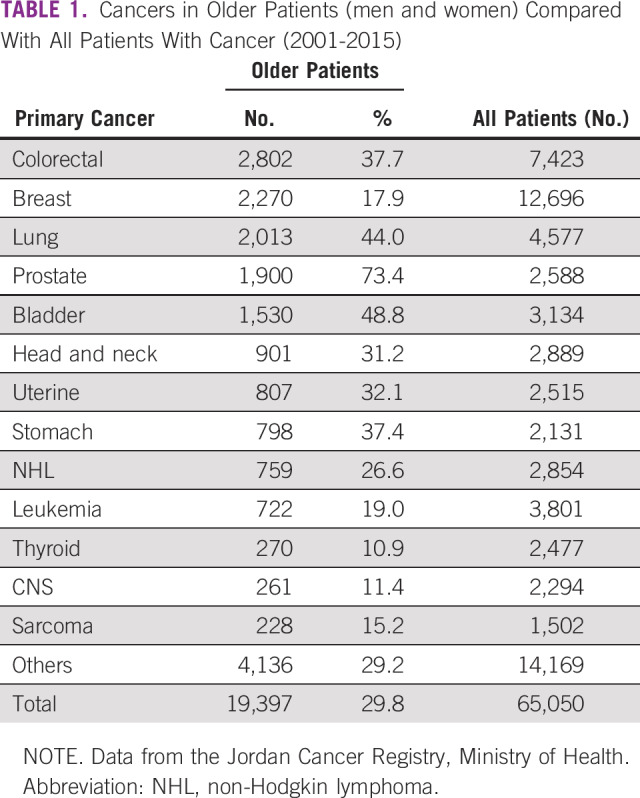
Cancers in Older Patients (men and women) Compared With All Patients With Cancer (2001-2015)

**FIG 3:**
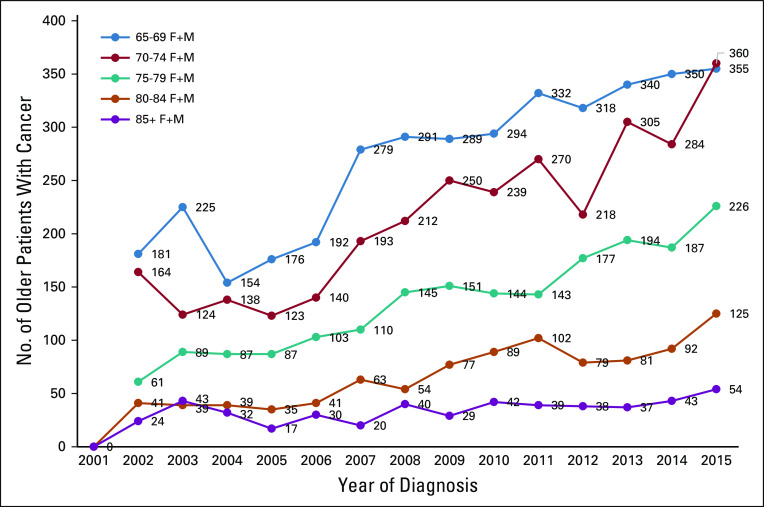
Cancer diagnoses among different age groups of older patients over the span of 15 years (2001-2015). F, female; M, male.

Since the introduction of the population-based national cancer registry in 1996, the total number of reported cancers increased from 3,412 in 2001 to 5,274 in 2015, a 55% increase. A greater increase was noted in older patients, in whom cancer diagnoses increased from 923 in 2001 to 1,632 in 2015, a 77% increase. Much of this increase was in patients age 65-69 years (Fig 3).

**FIG 4:**
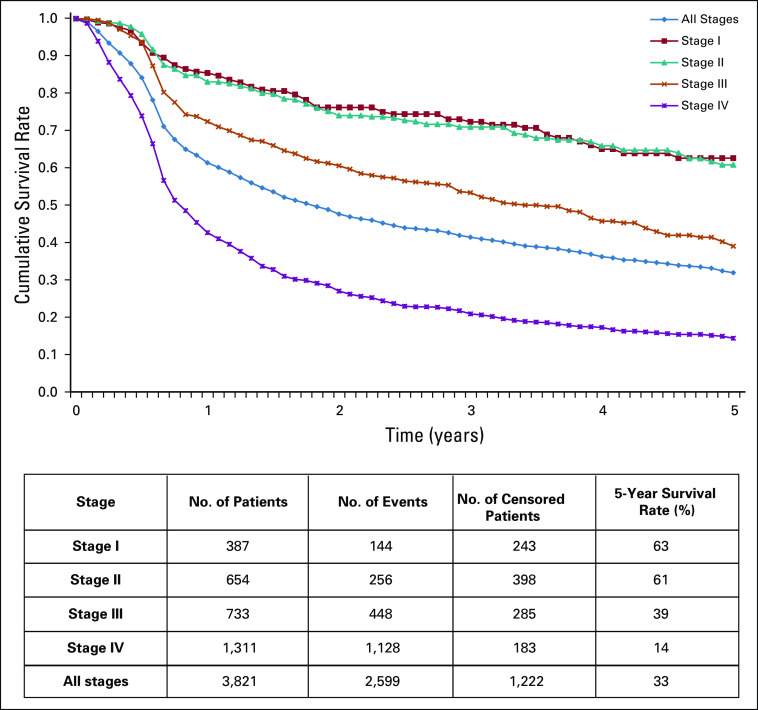
Five-year overall survival in older patients according to cancer stage.

Survival data were available for a total of 16,602 patients from the local cancer registry at King Hussein Cancer Center; among them, 3,821 (23%) were patients ≥ 65 years old. All patients had their pathology review, diagnosis, treatment, and follow-up at our institution. Five-year overall survival for the older patients was 33% overall but varied with disease stage (63% and 61% for stage I and II, respectively, *P* = .035; decreasing significantly to 39% for stage III and 14.4% for stage IV, *P* = .0001; Fig 4).

## DISCUSSION

It is evident that the witnessed increase in cancer diagnosis among older patients is much greater than the increase in younger age groups and even greater than the population growth itself. Much of this increase was seen among the younger groups (age 65-69 years) of older patients. Factors such as the late effects of a long history of smoking, obesity, lack of exercise, and modernization of our lifestyle may be important contributing factors.

With < 4% of our population age 65 years or older, our population is still a young one. Currently, 1 (9%) of 11 people globally are older than age 65, and by 2050, 1 (16%) in 6 people in the world will be older than age 65.^15^

Given the presented data, it is clear that the country and the region will encounter an increasing number of older patients; many of them will also be burdened with more comorbidities. This should direct us to invest more in prognostic and predictive indicators; some may help downscale interventions, including extensive surgery, aggressive chemotherapy, and even radiation therapy.

Participation of older patients in clinical trials is limited, and data regarding the efficacy and safety of treatment regimens in older patients, mostly dose reduced, are lacking.^16^ Age itself, underlying comorbidities, and psychosocial factors might result in underdosing of chemotherapy or radiotherapy or even reluctance to offer aggressive, potentially curative surgical procedures for such patients.

Survival curves are also interesting; overall survival of patients with stage I or stage II disease showed continued deterioration with no evidence of reaching a plateau, suggesting that such patients will continue to die, even with early-stage disease, mostly secondary to their comorbidities.

As the number of older patients with cancer in countries such as Jordan increases, oncologists will face more challenges managing patients with multiple chronic problems affecting their independence and making them more vulnerable to adverse outcomes. International guidelines recommend that all older patients with cancer undergo geriatric assessment to better address all their needs to improve their functional status and possibly improve their survival.^17^ Currently, such practice is not routinely done.

The patient’s preferences, at the core of the decision-making process in Western societies, are often dominated by family preferences in our region. A patient-centered approach is highly needed, especially so among older patients.

Our study is not without limitations. Our outcome data, although representing almost two thirds of patients with cancer in the country, are still from a single institution and do not represent the whole country. In addition, selection bias can be an additional factor; older patients with comorbidities and poor performance who are judged by their primary physicians not to be candidates for anticancer therapy might not be accepted to the center. In addition, demographic data reported by the JCR do not represent the current status and are at least 4 years old. Improving national cancer registry reporting of outcome data should improve planning and the decision-making process.

In conclusion, although < 4.0% of our local population is age 65 years or older, almost one third of the country’s cancer load occurs in this age group, and this portion is increasing at a rate faster than the population growth itself or cancers in other age groups. Preparing strategies, improving infrastructure, and creating programs that can deal in a comprehensive way with this increasing load of older patients affected by cancer, taking into consideration their high physical and psychosocial needs, should be a national health priority.
